# Structural and Catalytic Characterization of TsBGL, a β-Glucosidase From *Thermofilum* sp. ex4484_79

**DOI:** 10.3389/fmicb.2021.723678

**Published:** 2021-10-01

**Authors:** Anke Chen, Dan Wang, Rui Ji, Jixi Li, Shaohua Gu, Rong Tang, Chaoneng Ji

**Affiliations:** State Key Laboratory of Genetic Engineering, Institute of Genetics, School of Life Sciences, Fudan University, Shanghai, China

**Keywords:** *Thermofilum*, β-Glucosidase, glycoside hydrolase, catalytic site, thermostability, cellulase

## Abstract

Beta-glucosidase is an enzyme that catalyzes the hydrolysis of the glycosidic bonds of cellobiose, resulting in the production of glucose, which is an important step for the effective utilization of cellulose. In the present study, a thermostable β-glucosidase was isolated and purified from the *Thermoprotei Thermofilum* sp. ex4484_79 and subjected to enzymatic and structural characterization. The purified β-glucosidase (TsBGL) exhibited maximum activity at 90°C and pH 5.0 and displayed maximum specific activity of 139.2μmol/min/mg_zne_ against *p*-nitrophenyl β-D-glucopyranoside (*p*NPGlc) and 24.3μmol/min/mg_zen_ against cellobiose. Furthermore, TsBGL exhibited a relatively high thermostability, retaining 84 and 47% of its activity after incubation at 85°C for 1.5h and 90°C for 1.5h, respectively. The crystal structure of TsBGL was resolved at a resolution of 2.14Å, which revealed a classical (α/β)_8_-barrel catalytic domain. A structural comparison of TsBGL with other homologous proteins revealed that its catalytic sites included Glu210 and Glu414. We provide the molecular structure of TsBGL and the possibility of improving its characteristics for potential applications in industries.

## Introduction

The β-glucosidase (BGL) family (EC 3.2.1.21) comprise a wide range of enzymes that catalyzes the hydrolysis of the glycosidic bond to a nonreducing terminal beta-D-glucosyl residue, releasing glucose (Singh et al., 2016). BGLs play an important role in the cellulase system, which consists of endoglucanases (E.C.3.2.1.4), exoglucanases (E.C.3.2.1.91), and BGLs (E.C.3.2.1.21), and catalyze the last step in cellulose hydrolysis (Pei et al., 2012). BGLs are present in all kinds of organisms, including bacteria, archaea, and eukaryotes, and play several important roles in biological systems, including biomass conversion in microorganisms, glycolipid breakdown, and other ecological processes (Fleuri et al., 2009). Furthermore, these enzymes are used in various processes, including wine, food, and biofuel production and agriculture (Bhat, 2000; Agrawal et al., 2013; Maitan-Alfenas et al., 2014). However, the application of most natural enzymes in industries is limited by their nature and mild reaction conditions (Yenenler and Sezerman, 2016). Therefore, obtaining efficient and thermostable BGLs that can withstand industrial production has become the subject of recent research worldwide (Lorenz and Eck, 2005). High-thermostable BGLs have several advantages, including ease of purification by heat treatment and higher resistance to chemical denaturants. In addition, performing enzymatic reactions at high temperatures results in higher reaction rates, lower substrate viscosity, and fewer risks of microbial contamination (Vieille and Zeikus, 2001; Li et al., 2013). Therefore, identification of new sources of thermostable BGLs appears to be a promising strategy for biotechnological and industrial applications.

BGLs have various structures. Data hosted on the CAZy database reveal that BGLs are mainly distributed in GH families 1, 2, 3, 5, 9, 16, 30, 39, and 116, and its representative structure contains (β/α)_8_, β-jelly roll, and (α/α)_6_ (Henrissat and Davies, 1997; Cantarel et al., 2009). Members of the GH1 family have a (β/α)_8_-barrel domain structure that contains the active site, and the hydrolysis of the β-glycosidic bond is carried out *via* a catalytic mechanism that involves the action of two conserved glutamate residues that act as nucleophiles and proton donors (Sharma et al., 2019). Thermostable BGLs represent enzymes with unique structure–function properties, including an increased number of surface ion pairs, internal water molecules, and decreased surface area upon the formation of an oligomeric quaternary structure (Chi et al., 1999; Nakabayashi et al., 2014).

*Thermofilum* sp. ex4484_79 is a *Thermoprotei* isolated from hydrothermal deep-sea sediments (Dombrowski et al., 2017). Most enzymes in this archaea are thermophilic and exhibit resistance to high temperatures. In the present study, we isolated and purified a thermostable β-glucosidase from *Thermoprotei Thermofilum* sp. ex4484_79 (TsBGL), characterized its catalytic properties, and explored its crystal structure. Our results provide further insights into the potential industrial use of the key proteins involved in the enzymatic hydrolysis of cellulose.

## Materials and Methods

### Bacterial Strains, Plasmids, and Media

Plasmid pET22 was used as the vector for gene cloning and expression. *E. coli* strain DH5α (Transgen, China) was used as the host for cloning, and *E. coli* strain BL21 (DE3) plysS (Transgen, China) was used as protein expression. *E. coli* cells were grown at 37°C in LB medium containing 10g NaCl, 10g tryptone, and 5g yeast extract (Sangon Biotech, China) per liter at pH 7.0, and LB agar medium was added with 1.5–2.0% (w/v) agar.

### Sequence Analysis

Multiple alignment of amino acid sequences of homologs was performed using ClustalX v.2 program (Larkin et al., 2007). Second structure alignment was generated by the ESpript v.3.0 server (Robert and Gouet, 2014).

### Site-Directed Mutagenesis

*In vitro* site-directed mutagenesis of the TsBGL gene on plasmid pET22 was performed using KOD-Plus-Neo (TOYOBO, Shanghai, China). The primers used are listed (Table 1). All the mutants were confirmed by sequencing.

**Table 1:** Primers used to construct the mutants of TsBGL. The mutated bases were underlined in the site-directed PCR primers.

Mutants	Primers (5'to3')
E210A-F	GACTATTGGGTAACTTTTAACGCGCCCATGGTGGTTACGGAA
E210A-R	TTCCGTAACCACCATGGGCGCGTTAAAAGTTACCCAATAGTC
E414A-F	GGGAAACCACTGATTGTTACCGCGAACGGGATTGCAGATAGC
E414A-R	GCTATCTGCAATCCCGTTCGCGGTAACAATCAGTGGTTTCCC
M212W|A217L-F	GTAACTTTTAACGAGCCCTGGGTGGTTACGGAACTTGGCTACTTCCAGCCTGAG
M212W|A217L-R	CTCAGGCTGGAAGTAGCCAAGTTCCGTAACCACCCAGGGCTCGTTAAAAGTTAC

### Expression and Purification of the Recombinant TsBGL

The open reading frame (ORF) of TsBGL (GenBank: WP_010868057.1) and His6-SUMO tag was synthesized by GENEWIZ Company and cloned into pET22 plasmid between *NdeI* and *XhoI* sites to generate the pET22-SUMO-TsBGL expression plasmids (Supplementary Material). The wild type and mutants of pET22-SUMO-TsBGL expression plasmid were transformed into *E. coli* BL21 (DE3) plysS. Bacteria were grown at 37°C in LB medium containing 34mg·mL^−1^ chloramphenicol and 50mg·mL^−1^ ampicillin until the OD_600_ reached 0.6–0.8. Isopropyl-β-D-thiogalactopyranoside was added at a final concentration of 0.5mm to induce the expression of the recombinant protein. After induction at 25°C for 10h, the cells were collected by centrifugation at 5000×*g* for 5min at 4°C.

The collected cells were resuspended in lysis buffer (50mm Tris–HCl, 200mm NaCl, 10mm imidazole, pH 8.0; 10ml for every gram of the cell pellet), disrupted *via* two cycles of lysis in an ultra-low temperature cell sandblasting machine at a pressure of 1,200bar, and the cell debris were removed by centrifugation at 13000×*g* for 40min at 4°C. The supernatant was heated at 65°C for 30min and then centrifuged at 13000×*g* for 10min to remove the precipitate. After passing the supernatant through a HiTrapTM Ni-NTA column (GE Healthcare, United States) equilibrated with lysate, the resin was washed with a buffer (50mm Tris–HCl, 200mm NaCl, 50mm imidazole pH 8.0), and then, a linear elution gradient (50–500mm imidazole, 10ml) was used to elute TsBGL containing six His and SUMO tags. The tags were cleaved by incubating overnight with the Ulpl enzyme at 4°C as described in previous study (Malakhov et al., 2004). After the second pass through the Ni-NTA column, the target protein was detected by detagging. The target protein solution diluted with DEAE binding buffer (20mm Tris–HCl, pH 8.0) was passed through a DEAE column (GE Healthcare, United States) and linearly eluted using a DEAE elution buffer (20mm Tris–HCl, 500mm NaCl, pH 8.0; 0–500mm NaCl, 50ml; gradient elution). The recombinant protein was concentrated using a 30kDa centrifugal filter in combination with buffer exchange using 20mm Tris–HCl (pH 8.0). The concentration of TsBGL was determined using the Bradford method, and the purity of the protein was analyzed by electrophoresis on 12% SDS-PAGE gels. The concentrated TsBGL was frozen in liquid nitrogen and stored at-80°C.

### Biochemical Characterization of TsBGL

The enzymatic activity of wild-type TsBGL and mutants was determined using a microtiter plate method that measures the hydrolysis of *p*-nitrophenyl-β-D-glucopyranoside. For all enzymatic assays, the N-terminal His6-SUMO tag was removed using Ulp1 to prevent any interference with the enzymatic activity. The reaction mixture (100μl) contained 5μl of appropriately diluted enzyme and 95μl of 0.5mm *p*-nitrophenyl β-D-glucose (*p*NPGlc) in sodium acetate buffer (HOAc-NaOAc) pH 5.0. The reaction was stopped by addition of 100μl of 1M Na_2_CO_3_ after being incubated at 90°C for 10min. The nitrophenol released was quantified by measuring the absorbance at 410nm (Fusco et al., 2018). One unit of enzyme activity was defined as the amount of protein that produced 1.0μmol of the nitrophenol per minute under the standard assay conditions.

The behavior of the enzyme activity as a function of pH was studied by incubating the enzyme and substrate in several suitable buffer systems, i.e., HOAc-NaOAc (pH 3.0–6.0), citric acid-Na_2_HPO_4_ (pH 6.0–8.0), and HEPES-NaOH (pH 8.0–10.0), followed by measuring the activity under conditions of optimal reaction temperature (determined in preliminary experiments). The effect of temperature on enzymatic activity was evaluated by incubating the reaction at a temperature range of 55 to 100°C, and the activity was measured at the optimum pH.

The substrate specificity of TsBGL was determined using different cellulosic polysaccharides, oligosaccharides, and synthetic substrates and tested using the *p*-nitrophenol and GOD-POD (glucose oxidase-peroxidase) methods (McCleary and McGeough, 2015). The enzymatic activities were assayed against the carboxymethylcellulose (CMC) and oligosaccharides (cellobiose, lactose, maltose, and sucrose) under optimal reaction conditions for 10min, and the reaction was stopped by adding 100μl of 1M Na_2_CO_3_, and the concentration of glucose produced was estimated using the GOD-POD method with a commercially available kit. Enzymatic activities toward galactopyranoside and glucopyranoside were measured by using *p*-nitrophenyl-β-D-galactopyranoside (*p*NPGal) and *p*NPGlc as substrates, respectively.

Kinetic parameters using *p*NPGlc were determined by varying the concentration (0.1–6.0mm), and those for the natural substrate cellobiose were determined by varying the concentration (1–150mm) under optimal reaction conditions. The *K*_m_ and *V*_max_ values were calculated by nonlinear regression of the Michaelis–Menten plots with Graphpad Prism 8 (GraphPad Software, Inc., United States), and the apparent *k*_cat_ values were calculated by assuming all protein was active enzyme.

The influence of various metal ions and reagents on TsBGL-catalyzed reactions was evaluated by measuring the enzyme activity in the presence of KCl, MgCl_2_, MnCl_2_, FeCl_3_, CuCl_2_, CoCl_2_, NiCl_2_, BaCl_2_, SrCl_2_, ZnCl_2_, LiCl_2_, and EDTA (final concentrations, 1mm) and in response to n-dodecyl-N, N-dimethylamine-N-oxide (DDAO), n-octyl-β-D-glucoside, n-dodecyl-β-D-maltoside (DDM), trimethylamine N-oxide dihydrate (TMANO), Triton X-100, Tween 20, and SDS (all 1% solutions). All of the compounds were added to the buffer and substrate; then, the enzyme was added. Results were compared with those of the control sample that was not exposed to these chemicals (with a reference of 100%).

To evaluate thermal stability, the enzyme was incubated at different temperatures (85°C, 90°C, and 95°C) for different times (30, 60, and 90min) at the optimal pH. The residual activity of the enzyme was determined, considering the activity of the enzyme without pre-incubation was defined as 100%, and used to calculate the enzyme activity, expressed as a percentage of the enzyme activity without pre-incubation during incubation.

The product inhibition analysis test used *p*NPGlc as the substrate at the optimal pH and temperature, while adding glucose solutions of different concentrations to the reaction system, so that the final concentration in the system was 0.1, 0.25, 0.5, 1, 1.5, 2, and 2.5M, reacted for 10min, and the residual enzyme activity was measured after terminating the reaction.

### Crystallization and Data Collection

Commercial crystallization kits (Index and PEG/Ion from Hampton Research, Wizard Classic 1/2 and 3/4 block from Rigaku) were used to screen the preliminary results by sitting-drop vapor diffusion in 4×96 conditions at 16°C. The optimal crystallization conditions were 30% (v/v) 2-methyl-2,4-pentanediol, 100mm imidazole/hydrochloric pH 6.5, 200mm ammonium sulfate, and 10% (w/v) PEG 3350. The crystals were quickly immersed in an antifreeze solution composed of 80% stock solution and 20% glycerin and then immediately transferred to a nitrogen-cooled puck. Diffraction data were collected at a wavelength of 0.9792Å at-196°C. The MAR DTB detector was used to image the BL17U beamline of the Shanghai Synchrotron Radiation Facility (SSRF; Wang et al., 2018). The HKL-3000 software package was used to index, integrate, and zoom diffraction data (Minor et al., 2006).

### Structure Solution and Refinement

The structure was solved by molecular replacement with Phaser, as implemented in Phenix 1.15.2_3472, using the structural data for the BGL monomer from *Thermosphaera aggregans* (BGLTa; 41% sequence identity to TsBGL; PDB entry 1QVB; Chi et al., 1999) as the search model. Further iterations of refinement and model building were performed using the Phenix and Coot (Kwoun Kim et al., 2004). The atomic coordinates and structure factors have been deposited in the Protein Data Bank under the accession code 7F1N.

## Results

### Purification of BGL From *Thermofilum* sp.

**Figure 1:**
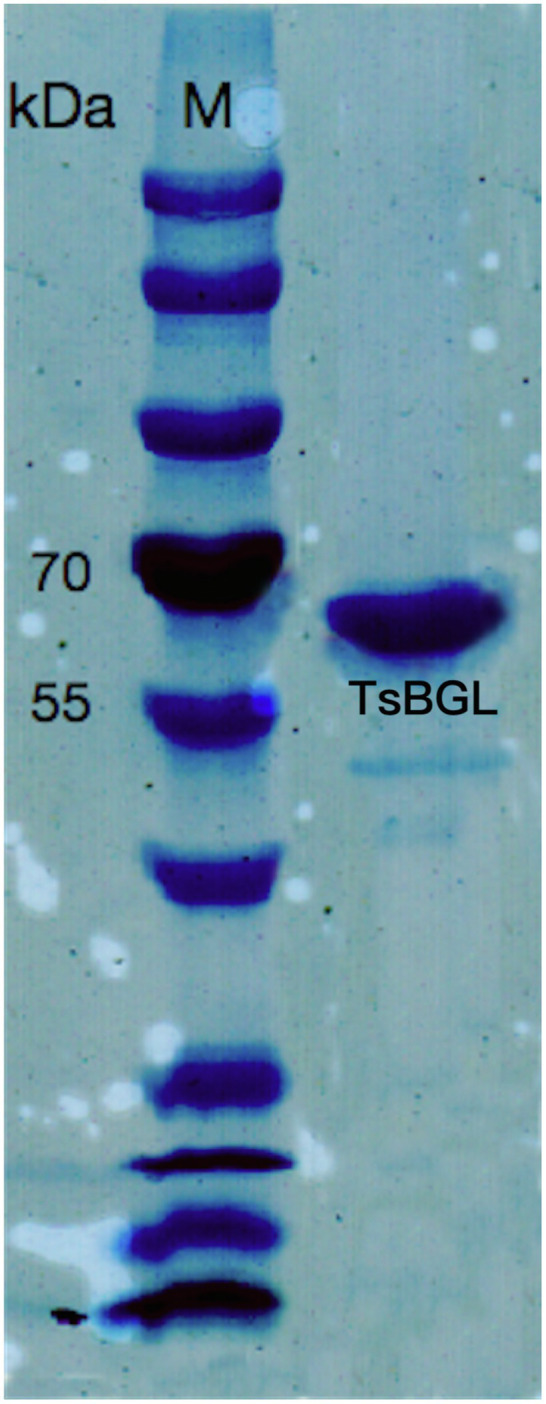
12% SDS-PAGE of TsBGL, eluted from the Ni column and DEAE column. The left lane was the molecular weight marker (labeled in kDa).

A putative ORF of 1716bp, encoding a 511-amino-acid protein with a theoretical molecular weight of 58.64kDa and pI of 5.56, was identified from the whole genome of *Thermofilum* sp. ex4484_79. A similarity search was performed with the BLAST program; the TsBGL protein was found to be a member the GH1 family. To perform the catalytic characterization of TsBGL, the recombinant TsBGL with an N-terminal His6-SUMO tag was expressed in *E. coli* BL21 (DE3) cells. The target protein TsBGL was purified to 95% homogeneity by Ni-NTA and DEAE columns. The protein concentration was approximately 15mg/ml (Figure 1).

### Characterization of TsBGL

**Figure 2:**
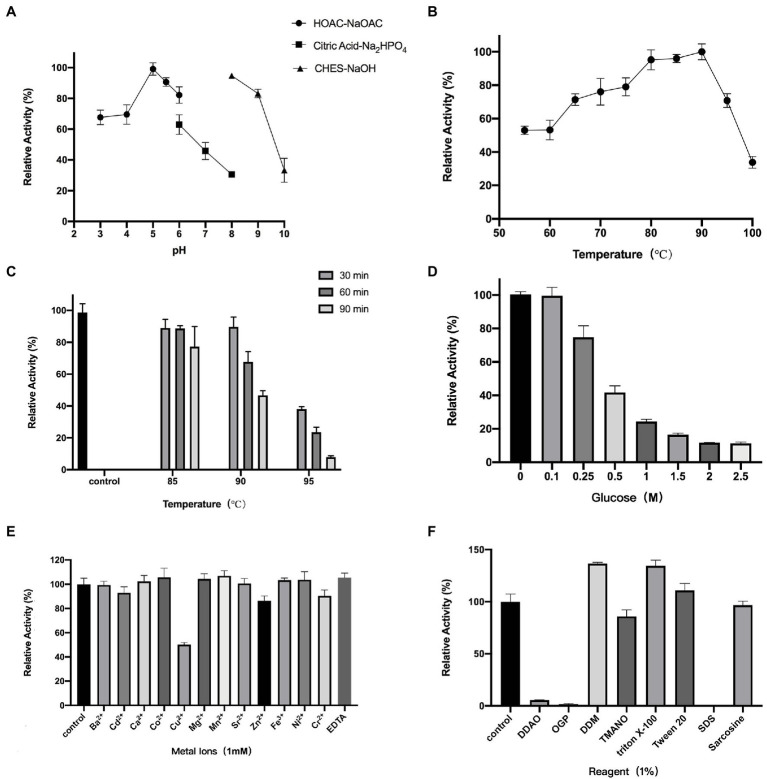
Enzymatic characterization of TsBGL. **(A)** Effects of PH on enzyme activity. The value obtained at PH 5.0 was taken as 100%. **(B)** Effects of temperature on enzyme activity. The value obtained at 90°C was taken as 100%. **(C)** Thermostability analysis. The value obtained without heat treatment was taken as 100%. **(D)** Effects of products (glucose) concentration on the activities. The value obtained without glucose in the reaction mixture was taken as 100%. **(E)** Effects of different metal ions on the enzymatic activity. The values obtained without ions in the reaction mixture were taken as 100%. **(F)** Effects of different detergents on the enzymatic activity. The values obtained without detergents in the reaction mixture were taken as 100%.

We first investigated the optimal temperature and pH dependence of TsBGL. The optimum reaction pH for TsBGL was 5.0 (Figure 2A). Under different buffer environments with the same pH, the enzyme activity differed. For example, at pH 6.0, the activity of TsBGL in the HOAc-NaOAc buffer was higher than that in the citric acid-Na_2_HPO_4_ buffer, and at pH 8, TsBGL activity in the CHES-NaOH buffer was higher than that in the citric acid-Na_2_HPO_4_ buffer. This may be attributed to the different ions having different electrostatic effects on the active center of the enzyme, thereby affecting the enzyme activity. TsBGL activity was increased between 60°C and 90°C, having a plateau at 80–90°C and reaching a maximal at 90°C (Figure 2B). TsBGL exhibited high thermostability, after incubating TsBGL at 85°C for 1.5h, 84% of the activity was retained, and at 90°C, TsBGL retained 47% of its activity for 1.5h (Figure 2C). In addition, the effect of glucose on TsBGL was also investigated (Figure 2D); using the lithon method (Kalliokoski et al., 2013) to calculate the glucose concentration that inhibits TsBGL enzyme activity by 50%, the IC50 is 0.35M.

Among the different substrates tested, TsBGL exhibited the best hydrolyzing capacity against cellobiose and *p*NPGlc. Lactose was hydrolyzed at 52.6% of cellobiose, and *p*NPGal was hydrolyzed at 75.4% of *p*NPGlc. In addition, no observable activity against maltose, sucrose, and CMC as substrates was detected. The kinetic parameters obtained with *p*NPGlc, *p*NPGal, cellobiose, and lactose as substrates under the optimized enzymatic conditions (pH 5.0, 90°C) showed that TsBGL exhibited a typical Michaelis–Menten behavior with half-saturation constant (*K*_m_), maximum velocity (*V*_max_), and catalytic constant (*k*_cat_) values of 0.617mm, 139.2μmol/min/mg_enz_ and 136.05s^−1^, respectively, for *p*NPGlc, 6.24mm, 24.3μmol/min/mg_enz_ and 23.8s^−1^, respectively, for cellobiose, 1.05mm, 127.4μmol/min/mg_enz_ and 124.5s^−1^, respectively, for *p*NPGal, and 38.1mm, 7.59μmol/min/mg_enz_ and 7.46s^−1^, respectively, for lactose (Table 2).

**Table 2:** Effects of the β-glucosidase on different substrates.

Substrates	Relative activity (%)	*K*_m_ (mm^−1^)	*V*_m_ (μmol/min/mg_zen_)	*k*_cat_ (s^−1^)	*k*_cat_/*K*_m_ (mm^−1^s^−1^)
**Chromogenic substrate (0.5mm)**					
*p*-Nitrophenyl β-D-glucopyranoside (*p*NPGlc)	99.7 ± 1.8	0.617	139.2	135.1	220.5
*p*-Nitrophenyl-β-D-galactopyranoside (*p*NPGal)	75.4 ± 3.1	1.054	127.4	124.5	118.1
**Oligosaccharides (5mm)**					
Cellobiose (β-1,4)	100.5 ± 8.9	6.24	24.3	23.8	3.81
Lactose (β-1,4)	52.6 ± 6.5	38.1	7.59	7.46	0.196
Maltose (α-1,4)	ND				
Sucrose (α-1,2)	ND				
CMC (1%)	ND				

*Values presented are the means ± SD, and the SD was calculated from three independent reaction tubes using the same purified BGL*

Furthermore, the effects of metal ions—at concentrations of 1mm—and reagents—at concentrations of 1%—on TsBGL activity were investigated (Figures 2E,F). Cu^2+^ strongly inhibited TsBGL activity resulting in only 50% activity; Cu^2+^ has been reported to be a strong inhibitor of most BGLs, suggesting that it is a potent oxidative agent and can inhibit the catalytic activity of cellulase (Tejirian and Xu, 2010; Sorensen et al., 2013; Crespim et al., 2016; Liew et al., 2018). However, no obvious effect was detected with the remaining eleven metal ions and EDTA. Among the seven detergents, SDS completely inhibited the TsBGL activity; it is speculated that SDS destroys the non-covalent bonds inside the protein and causes the loss of its natural structure and function. OPG and DDAO also significantly inhibited the TsBGL activity (1.6 and 5.5% residual activity at a concentration of 1%). The activity of TsBGL was remarkably increased in the presence of DDM and Triton X-100 (increase of 36 and 34% relative activity, respectively). TMANO, Tween 20, and sarcosine had little effect on the activity of TsBGL. This information regarding the physicochemical characteristics of cellulolytic enzymes is an important step in the production of enzymes with industrial applications.

### Overall Structure of TsBGL

**Figure 3:**
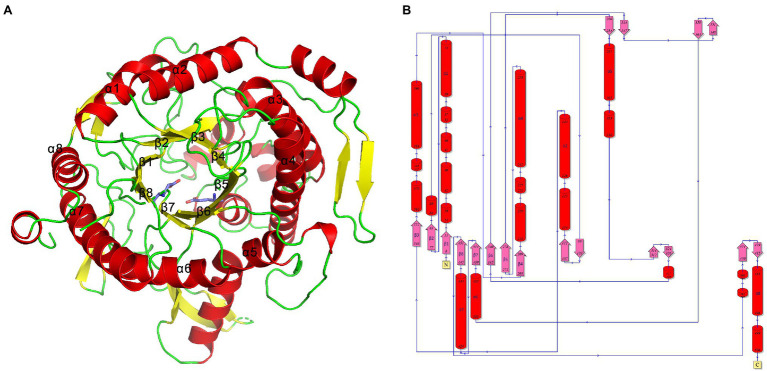
**(A)** Cartoon representation of TsBGL with the label secondary structure elements, α-helices were shown in red, and β-strands were shown as yellow arrows. **(B)** Topology diagram of TsBGL. The α-helices and β-strands are red cylinder and pink arrow, respectively.

The TsBGL crystal belonged to the space group P12_1_1 and diffracted to a resolution of 2.14Å. Its unit cell parameters were *α* =90°, *γ* =98.508°, *β* =90°, *a* =76.72Å, *b* =62.21Å, and *c* =112.45Å. The Matthews coefficient was 2.31Å^3^ Da^−1^, and the solvent content was 46.7%. Data collection and final refinement statistics are shown in Table 3. The asymmetric unit of the crystal structure consisted of two protein molecules. From the appearance point of view, TsBGL is composed of two large loops: the outer α-helix loop and the inner β-helix loop. The overall view indicates that TsBGL has a classic (β/α)_8_-barrel domain structure. These classic (β/α)_8_ barrels were first discovered in triose phosphate isomerase in 1975 and are therefore referred to as a TIM barrel (Banner et al., 1975). This classic TIM barrel structure is observed in all known members of the glycoside hydrolase family 1. The structure of TsBGL comprised 18 β-strands, 37 β-turns, and 22 α-helices. There were three types of 18 β-strands, 10 parallel, and 8 anti-parallel. The central (β/α)_8_ barrels consisted of eight parallel β-strands, namely β1 (Phe8-Ser13), β2 (Asn77-Ile83), β3 (Lys146-Asn152), β4 (Asp203-Asn209), β5 (Tyr271-Ile278), β6 (Trp335-Tyr340), β7 (Pro409-Glu414), and β8 (Asn445-His451), surrounded by eight α-helices, namely, α1 (Asn59-Ile74), α2 (Asn126-Arg143), α3 (Lys181-Gly199), α4 (Asn232-Asp258), α5 (Arg287-Asn302), α6 (Pro393-Glu405), α7 (Ile424-Lys444), and α8 (Arg483-Lys496; Figure 3).

**Table 3:** Data collection and structure refinement statistics.

Items	TsBGL
**Data collection**	
Diffraction source	Beamline BL17U, SSRF
Wavelength (Å)	0.9792
Temperature (K)	100
Detector	MAR DTB
Resolution range (Å)	111.21–2.14 (2.20–2.14)
Space group	P1211
a, b, c (Å)	76.72, 62.21, 112.45
α, β, γ (°)	90, 98.508, 90
Total no. of reflections	362,825 (20049)
No. of unique reflections	56,997 (3988)
Multiplicit	6.4 (5.0)
<I/σ(I)>	3.6 (2.2)
Completeness (%)	98.2 (94)
Rmerge	0.437 (0.503)
CC1/2	0.857 (0.709)
Solvent content (%)	46.7
Mosaicity (°)	2.31
**Refinement**	
No. of protein chains	2
No. of non-hydrogen atoms	8,599
R-work	0.2097 (0.2291)
R-free	0.2526 (0.2717)
RMS bonds (Å)	0.003
RMS angles (°)	0.63
Ramachandran favored (%)	97
Ramachandran allowed (%)	2.9
Ramachandran outliers (%)	0.1
Clashcore	5.90
Wilson B-factor	24.75
Average B-factor	25.97
Protein	25.67
Solvent	31.23
PDB accession code	7F1N

*Values in parentheses are for the outer shell*.

### Catalytic Site Analysis

**Figure 4:**
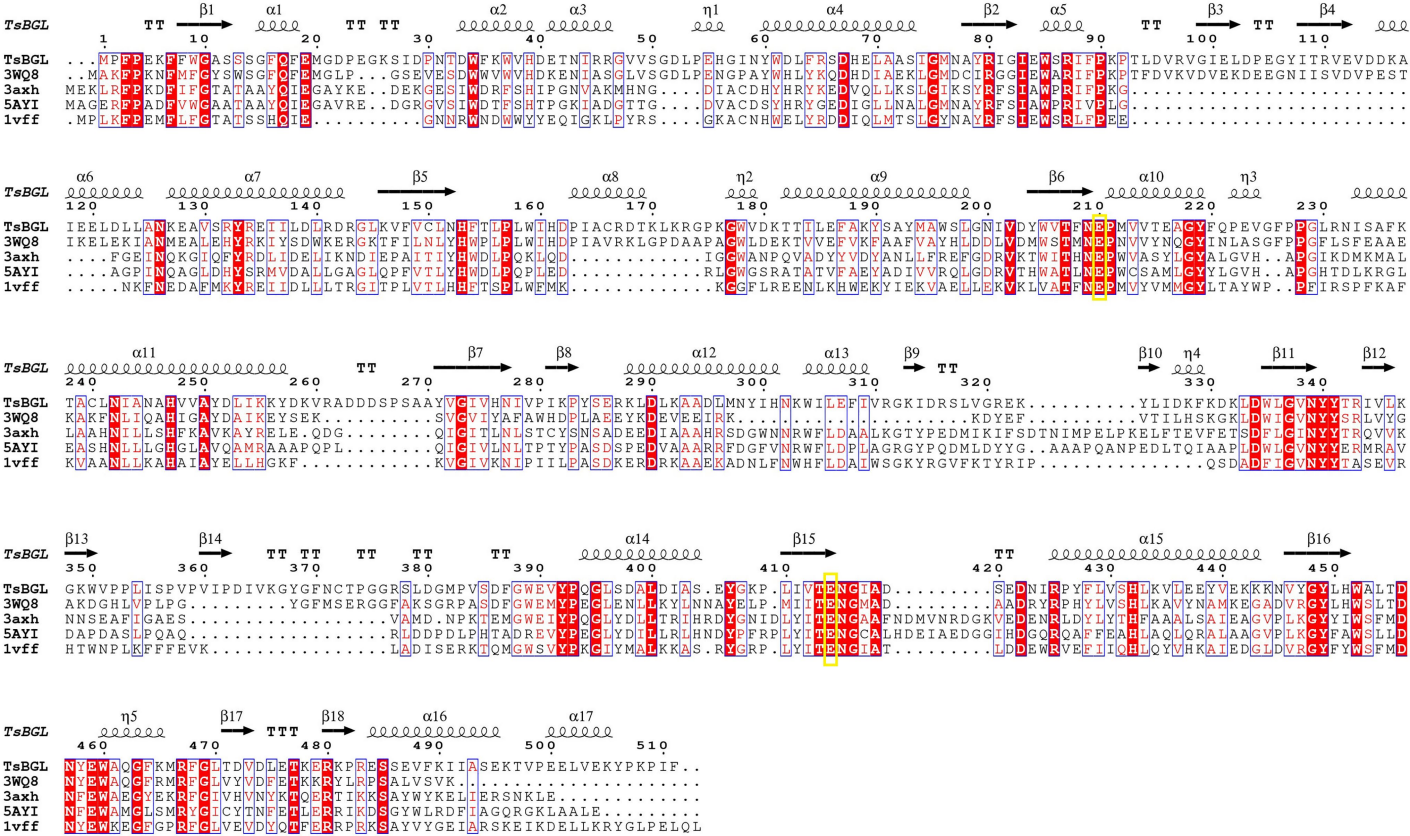
Multiple sequence alignment of TsBGL with other GH1 family β-glucosidases (BGLs), 3AXH from *Clostridium cellulovorans*, 3WQ8 from *Pyrococcus furiosus*, 1VFF from *Pyrococcus horikoshii*, and 5AYI isolated from a compost metagenome.

**Figure 5:**
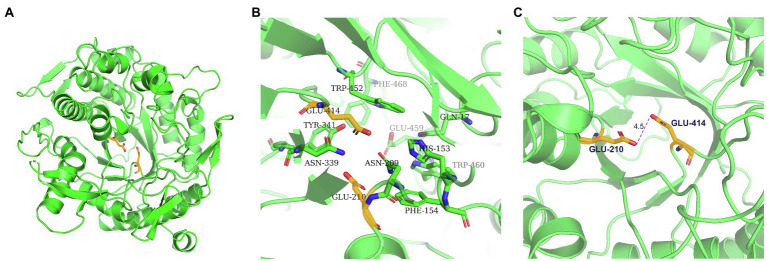
Structure of the TsBGL active site. **(A)** The overall structure of TsBGL adopts a typical (β/α)_8_TIM-barrel fold. The side chains of the two important glutamates in the active-site of TsBGL are shown as stick models. **(B)** The active site regions of TsBGL. **(C)** The closest distance between the catalytic residues (Oε2 atom of Glu210 and Oε1 atom of Glu414).

GH1 BGLs retain the retention mechanism involving the 4- and 7-terminal glutamate of the β chain; therefore, they are called “4/7 superfamily” enzymes (Pickersgill et al., 1998). Structure-based sequence alignment on PDB showed that β-glucosidase from different sources have conserved structures. We selected four proteins, all of which have structured in PDB, and are about 20–40% homologous to TsBGL in sequence, and the homologousness of the four proteins to each other cannot be too high. 3AXH from *Clostridium cellulovorans*, 3WQ8 from *Pyrococcus furiosus*, 1VFF from *Pyrococcus horikoshii*, and 5AYI isolated from a compost metagenome (Figure 4). We found that GH1 BGLs have a catalytic acid/base glutamate and catalytic nucleophile glutamate in the highly conserved TENEP and TENG motifs (Henrissat and Davies, 1997; Rye and Withers, 2000; Chuankhayan et al., 2007). In TsBGL, Glu210 and Glu414 served as the putative catalytic residues. The active center of TsBGL was surrounded by aromatic groups and polar residues, including Gln17, His153, Phe154, Asn209, Glu210, Asn339, Tyr341, Glu414, Trp452, Glu459, Trp460, and Phe468. Glu414 was located at the end of β-strand 7, Glu210 was located behind Asn209, which was the end of β-strand 4, Glu210 is a proton donor, Glu414 is a catalytic nucleophile, and the closest distance between Glu210 and Glu414 was 4.5Å, which matched the catalytic characteristics of GH1 BGLs (Figure 5).

Moreover, multiple grooves were observed on the surface of the GH1 BGL family protein. Research indicates that the largest and deepest cleft can be presumed to be substrate-binding channels. This cleft was approximately 27Å deep from the surface of the molecule to the inside, which was sufficient to accommodate glycoside and disaccharide molecules (Chuenchor et al., 2008). Many grooves were also observed on the surface of TsBGL, and the largest and deepest cleft was considered the key location for enzymatic reactions.

### Mutation of Catalytic and Gateway Amino Acid Residues

**Figure 6:**
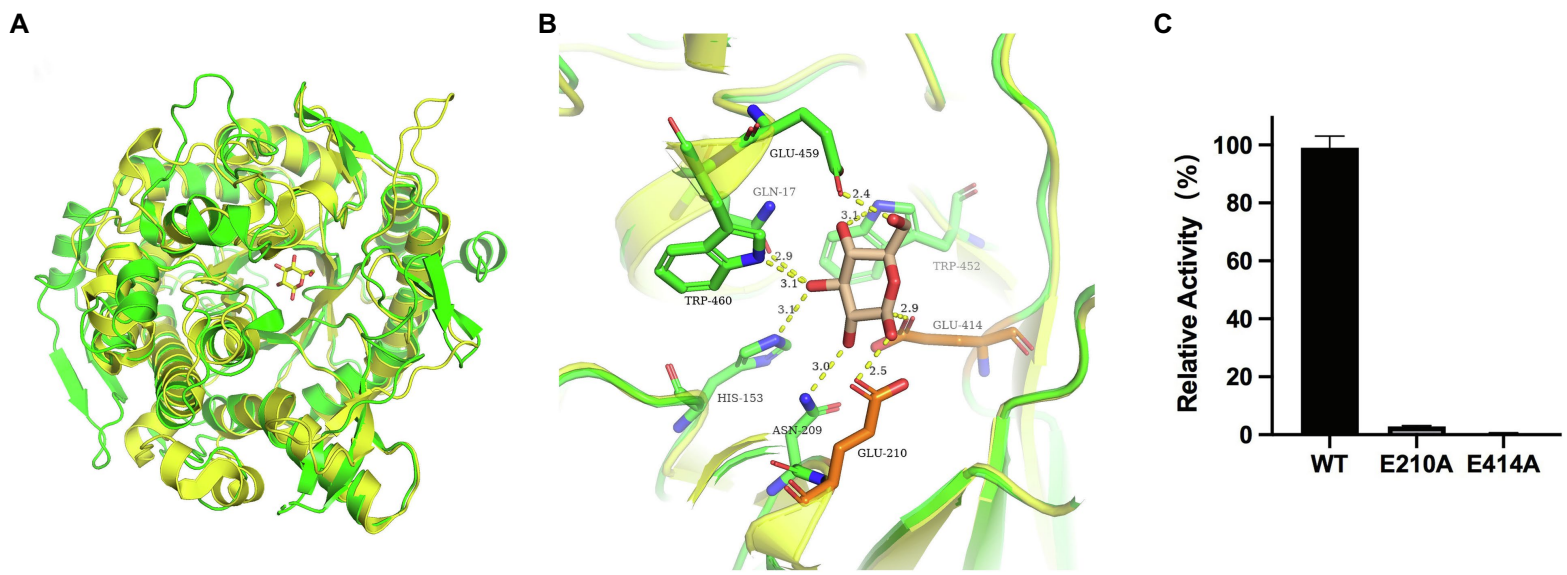
Structural comparison of GH1 at subsite-1. **(A)** Structural comparison of TsBGL (green) with Td2F2 (yellow, PDB: 5AYI, RMSD=0.909. **(B)** Superposition of the active sites of TsBGL (green) and Td2F2 (yellow). The product of glucose was shown in wheat sticks. **(C)** Hydrolysis activity of TsBGL and active site mutants using *p*NPGlc as the substrate.

**Figure 7:**
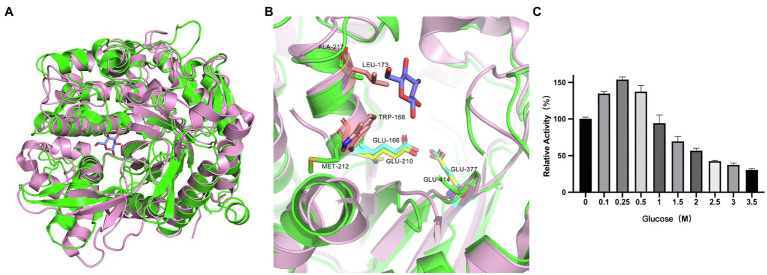
Analysis of active-site accessibility. **(A)** Structural comparison of TsBGL (green) with HiBG (pink, PDB: 4MDP, RMSD=1.316). **(B)** The stick representation of the two gatekeeper residues (Trp168 and Leu173) in HiBG substituted with a Met212 and Ala217 in TsBGL. **(C)** Effects of glucose concentration on the activities of M212W|A217L mutant. The value obtained without glucose in the reaction mixture was taken as 100%.

According to the catalytic site analysis, we generated the E210A and E414A mutations to verify the putative catalytic residues in TsBGL. We found that E210A and E414A mutations significantly decreased enzymatic activity (Figure 6C). Based on the structure of HiBG (PDB: 4MDP), a highly glucose-tolerant GH1 BGL from Humicola insolens (de Giuseppe et al., 2014), the two HiBG residues Trp168 and Leu173 were considered gatekeepers involved in glucose tolerance, and these two residues were not conserved in TsBGL and were replaced by Met212 and Ala217, respectively (Figures 7A,B). To improve glucose tolerance of TsBGL, site-directed mutagenesis was performed. The M212W|A217L mutant resulted in significantly increased glucose tolerance, up to 6-fold, and showed an activity of 50% in the presence of 2.14M glucose (Figure 7C).

## Discussion

BGL is an essential enzyme in the efficient hydrolysis of cellulosic biomass, as it catalyzes the conversion of cellobiose to glucose and relieves cellobiose inhibition of cellobiohydrolases and endoglucanases (Srivastava et al., 2019). In this study, we cloned the gene encoding BGL in *Thermofilum* sp. ex4484_79. According to amino acid sequence analysis, although the ORF encoding TsBGL is annotated as a β-galactosidase in Genbank, the specificity constant *k*_cat_/*K*_m_ values of TsBGL catalyzing different substrates *p*NPGlc, cellobiose, *p*NPGal, and lactose are 220.5mm^−1^ s^−1^, 3.81mm^−1^ s^−1^, 118.1mm^−1^ s^−1^, and 0.196mm^−1^ s^−1^, respectively. This indicates that TsBGL has a stronger affinity with *p*NPGlc and cellobiose (Table 2).

TsBGL had the same (β/α)_8_-barrel domain structure as other members of the GH1 family. In addition, based on homology, the mechanism underlying the effects of TsBGL is expected to be the same as other retained glycoside hydrolases of GH1. The hydrolysis reaction is carried out by two amino acid residues (usually glutamic acid and aspartic acid) separated by approximately 5Å, which function as a nucleophile and a proton donor, respectively (Rye and Withers, 2000). The predicted amino acid site of TsBGL was superimposed with that of Td2F2 (PDB: 5AYI, RMSD=0.909), a glucose binder in the GH1 family (Matsuzawa et al., 2016). Structural overlay in the depths of the fissure of TsBGL revealed the distance between an oxygen atom of Glu414 and anomeric carbon C1 of the glucose residue based at subsite-1 to be 2.9Å. This indicated that the Glu414 residue can act as a pronuclear reagent for pronuclear attacks on anomeric carbon C1 (Figures 6A,B). According to the catalytic retention mechanism (Vuong and Wilson, 2010), in the first reaction step, the Glu210 residue is an acid, providing a catalytic departure group, and the oxygen atom of the Glu414 residue and anomeric carbon C1 of glucose residue at subsite-1 form C-O bonds. Meanwhile H+ and β-1,4 glucoside bonds break, forming a glyco-enzyme intermediate, to complete the glucose-based process. In the second reaction step, the Glu210 residual base with a negative charge is a catalytic alkali that sequestered the protons of the water molecules and then activates the pronuclear reagents to hydrolyze the glycosyl-enzyme intermediates, thereby completing the desaccharization process. To verify the putative catalytic residues in TsBGL, site-directed mutagenesis was performed. The substitution of either Glu210 or Glu414 with alanine significantly decreased the enzymatic activity (Figure 6C). These results suggested that Glu210 and Glu414 act as nucleophiles and proton donors, respectively, and play vital roles in determining the enzyme activity. Moreover, other residues appeared to be glucose-binding residues in Td2F2 and were completely conserved in TsBGL, including the Gln17, His153, Asn209, Asn339, Tyr341, Trp452, Glu459, and Trp460 residues, which in the fissure might be bound to the appropriate position by a hydrogen bond network that is dissipated in time after completion of the reaction (Barrett et al., 1995).

Thermostable cellulolytic enzymes, especially β-glucosidase, are valuable in industrial applications because saccharification is often carried out at 50°C for extended periods (sometimes more than 100h; Hodge et al., 2009). Consequently, robust thermostability improves fermentation yields, reduces contamination risk, requires less energy consumption, reduces production costs, and enhances enzyme activity and solubility of the reactants and products (Haki and Rakshit, 2003; Liu et al., 2012). The TsBGL exhibited the highest activity at 90°C and retained approximately 50% of its activity after incubation at 90°C for 1.5 h (Figures 2B,C), which were better than that of most BGLs from other thermophilic bacteria. For example, BGL from *Hungateiclostridium thermocellum* exhibits maximum activity at 65°C and retain 50% of its activity after incubation at 60°C for 1h, that from *Thermoanaerobacterium thermosaccharolyticum* DMS 571 exhibits maximum activity at 70°C and retain 50% of its activity after incubation at 60°C for 2h, and that from *Caldicellulosiruptor saccharolyticus* DSM 8903 exhibits maximum activity at 70°C and retain 50% of its activity after incubation at 70°C for 24h. (Hong et al., 2009; Pei et al., 2012; Sharma et al., 2019). In addition, the comparable thermostability of BGLs from hyperthermophilic archaeal has been reported. BGL from *Thermotoga petrophila* exhibits the highest activity at 90°C and retains 50% of its activity after incubation at 90°C for 1.5h that from *Thermotoga neapolitina* exhibits the highest activity at 95°C and retains 50% of its activity after incubation at 100°C for 3.6h and that from *Thermus nonproteolyticus* exhibits the highest activity at 90°C and retains 50% of its activity after incubation at 90°C for 2.5h (Xiangyuan et al., 2001; Park et al., 2005; Haq et al., 2012). Meanwhile, some exceptionally hyperthermostable BGLs have been cloned. BGL from *Thermotoga maritima* was determined to be stable for 12.6 h at 100°C, and that from *Pyrococcus furiosus* was found to be stable for 85 h at 100°C. (Voorhorst et al., 1995; Mehmood et al., 2014; Table 4). The TsBGL also has excellent performance at the optimal reaction temperature and thermostability compared to other BGLs from hyperthermophilic archaeal.

**Table 4:** Compared enzyme activity between TsBGL with other BGLs from hyperthermophilic archaeal and thermophilic bacteria.

Strain	*K*_m_ (mm)		*V*_m_ (μmol/min/mg_enz_)		Glucose for IC50 (M)	Optimal temperature	Thermostability^b^	Ref.
	*p*NPGlc	cellobiose	*p*NPGlc	cellobiose				
*Thermofilum* sp. ex4484_79	0.617	6.24	139.2	24.3	0.35	90°C	90°C for 1.5h	This article
*Thermotoga petrophila*	2.8	ND^a^	42,700	ND	ND	90°C	90°C for 1.5h	Haq et al., 2012
*Thermotoga neapolitina*	0.89	66.62	701.6	408.3	ND	95°C	100°C for 3.6h	Park et al., 2005
*Thermus nonproteolyticus*	0.9	ND	112	ND	ND	90°C	90°C for 2.5h	Xiangyuan et al., 2001
*Thermotoga maritima*	0.56	ND	238	ND	ND	80–100°C	100°C for 12.6h	Mehmood et al., 2014
*Pyrococcus furiosus*	0.15	20	700	470	0.3	100°C	100°C for 85h	Voorhorst et al., 1995
*Thermotoga thermarum* DSM 5069T	0.59	35.3	142	19	1.5	90°C	95°C for 1h	Zhao et al., 2013
*Thermotoga naphthophila* RKU-10T	1.5	7.76	297,000	263	1.2	95°C	100°C for 2h	Fatima et al., 2016
*Thermococcus* sp.	7.6	16.48	112	105	4	80°C	78°C for 4h	Sinha and Datta, 2016
Thermoanaerobacterium thermosaccharolyticum	0.63	7.9	64	120	0.6	70°C	60°C for 2h	Pei et al., 2012
*Caldicellulosiruptor saccharolyticus*	0.67	ND	13	16.2	ND	70°C	70°C for 24h	Hong et al., 2009
Hungateiclostridium thermocellum	1.6	7.6	82.6	180	0.75	65°C	60°C for 1h	Sharma et al., 2019
Thermoanaerobacterium aotearoense	0.66	25.45	180.6	740.5	0.8	60°C	50°C for 3.3h	Yang et al., 2015

a *ND not determined*.

^b^*50% residual enzyme activity was retained after incubation*.

In general, the stability of a protein is related to its amino acid composition. Research has shown that the optimum temperature of enzymes increases as the percentage of acidic amino acids increases (Voorhorst et al., 1995; Godde et al., 2005). Compared with enzymes from mesophilic bacteria, enzymes from thermophilic bacteria contain more acidic amino acid residues (especially glutamate), a phenomenon that is thought to be responsible for the thermostability of these enzymes (Singh and Hayashi, 1995). TsBGL was found to contain 15% acidic amino acids (37 glutamic acid residues and 39 aspartic acid residues), and BGPh from *Pyrococcus horikoshii* OT3 (Akiba et al., 2004), BGTa from *Thermosphaera aggregans* M11TL (Chi et al., 1999), and BGSs from *Sulfolobus solfataricus* (Aguilar et al., 1997) have 12.5, 12.8, and 12.6% acidic amino acids, respectively, a percentage that is much higher than the average percentage of acidic amino acids in mesophilic glucosidases, like CBG, from *white clover* (Barrett et al., 1995), which has 8.7% acidic amino acids. Moreover, TsBGL has approximately 37% of its charged residues involved in ion-pairs, whereas for BGPh, BGTa, and BGSs, 35.2, 41, and 43.2% of its charged residues form ion pairs, respectively. These charged residues are conducive to the formation of ion pairs, which is one of the reasons for the thermostability of thermophilic proteins. Hydrophobic interactions inside protein molecules are another important factor for stabilizing the protein structure. TsBGL contains 55 aromatic amino acids, which together account for 11% of the protein sequence. On average, the non-thermostable BGLs (randomly drawn from the NCBI protein database) comprise 9% aromatic acid content in terms of the protein sequence (Jabbour et al., 2012).

BGLs are key enzymes for converting cellulose to glucose and exhibit a feedback inhibition effect on the reaction. Therefore, search for BGLs with a high tolerance to glucose is beneficial for the conversion of cellulose. However, most of the glucose-tolerant BGL belonging to GH1 family were reported, which showed IC50 values varying between 0.8 and 4.0M, but the thermostabilities of these enzymes were typically poor. For example, the half-lives of glucose-tolerant BGL from *Thermoanaerobacterium aotearoense*, *Aspergillus oryzae*, and *Candida peltata* were 3.3, 4, and 0.5h at 50°C, respectively (Saha and Bothast, 1996; Riou et al., 1998; Yang et al., 2015). To our knowledge, only three BGLs from hyperthermophilic archaea exhibit both strong glucose tolerance and excellent thermostability. BGL from *Thermotoga naphthophila* RKU-10T has half-lives of 2h at 100°C and IC50 value of 1.2M, BGL from *Thermotoga thermarum* DSM 5069T has half-lives of 1h at 95°C and IC50 value of 1.5M, and BGL from *Thermococcus* sp. has half-lives of 4h at 78°C and IC50 value of 4M (Zhao et al., 2013; Fatima et al., 2016; Sinha and Datta, 2016; Table 4). TsBGL showed an activity of 50% in the presence of 350mm glucose, indicating that it can circumvent product inhibition, but its tolerance is lower than that of other glucose-tolerant enzymes. It was reported that the two HiBG residues Trp168 and Leu173 were considered gatekeepers involved in glucose tolerance and contribute to reducing the inhibitory effect of glucose by imposing space constraints (de Giuseppe et al., 2014). However, these two residues were not conserved in TsBGL and were replaced by Met212 and Ala217, respectively (Figures 7A,B). These two TsBGL residual side chains are smaller and could make fewer hydrophobic interactions. Therefore, the size of the subsite +2 is increased, thereby increasing the possibility of glucose entering subsite-1, which leads to product inhibition. To improve glucose tolerance of TsBGL, site-directed mutagenesis was performed. The M212W|A217L mutant had significantly increased glucose tolerance, up to 6-fold, and showed an activity of 50% in the presence of 2.14M glucose, which was better than BGLs from *Thermotoga thermarum* DSM 5069T and *Thermotoga naphthophila* RKU-10T, only lower than BGL from *Thermococcus* sp., but the thermostability of *Thermococcus* sp. was not as good as ours. The TsBGL mutant provides a new β-glucosidase with both excellent thermostability and high glucose tolerance, and has potential prospects for industrial applications.

Moreover, high enzyme activity of BGLs is also required for the enzymatic hydrolysis of cellulose. The maximum velocity (*V*_max_) of TsBGL was 139.2μmol/min/mg_enz_ for *p*NPGlc and only 24.3μmol/min/mg_enz_ for cellobiose. We found that cellobiose had a lower *V*_max_ than *p*NPGlc, which conformed to a model for substrate preference (Nam et al., 2010). The S1 substrate-binding site of BGLs had a rigid structure. The nitrophenyl group of *p*NPGlc was located exactly in the active center of the S1 pocket. However, the second glucose of cellobiose used the rotation of the σ-bonds of glucosides to alter the composition of the substrate and then bind to the S1 substrate-binding site.

## Conclusion

We used a rational approach to investigate the biochemical characteristics of TsBGL and to obtain a detailed three-dimensional structure. These findings can provide a structural and theoretical basis for the study of new industrial enzymes in biotechnology applications.

## Data Availability Statement

The datasets generated for this study can be found in the structure of TsBGL was deposited in Protein data bank (PDB, http://www.rcsb.org/). The PDB ID is 7F1N.

## Author Contributions

AC carried out the experiments of biochemical characterization. DW carried out the experiments of crystallization. RJ contributed to the structural analysis. SG and RT guided the experiments of protein expression and purification. JL guided the analysis of the data. CJ designed the overall study and drafted the manuscript. All the authors read and approved the final manuscript.

## Funding

This research was funded by the grants from the National Key Research and Development Program of China (no. 2016YFA0500600) and Science and Technology Research Program of Shanghai (no. 19DZ2282100).

## Conflict of Interest

The authors declare that the research was conducted in the absence of any commercial or financial relationships that could be construed as a potential conflict of interest.

## Publisher’s Note

All claims expressed in this article are solely those of the authors and do not necessarily represent those of their affiliated organizations, or those of the publisher, the editors and the reviewers. Any product that may be evaluated in this article, or claim that may be made by its manufacturer, is not guaranteed or endorsed by the publisher.
