# Supplementary figures and images for: Structural and Catalytic Characterization of TsBGL, a β-Glucosidase From Thermofilum sp. ex4484_79
Source: Front Microbiol. 2021 Oct 1;12:723678. doi: 10.3389/fmicb.2021.723678 (PMC8517440; doi:10.3389/fmicb.2021.723678)

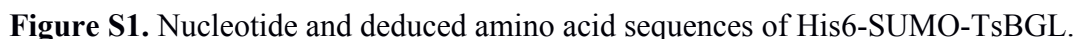

Supplement: Supplementary file 1 [file Image_1.pdf]
